# Extrapulmonary small cell sarcinoma: involvement of the brain without evidence of extracranial malignancy by serial PET/CT scans

**DOI:** 10.1186/1477-7819-6-102

**Published:** 2008-09-25

**Authors:** Christopher N Hueser, Nghi C Nguyen, Medhat Osman, Necat Havlioglu, Anjali J Patel

**Affiliations:** 1 Department of Internal Medicine, Division of Hematology and Oncology, St. Louis University Hospital, St. Louis, MO 63110, USA; 2 Department of Nuclear Medicine, St. Louis University Hospital, St. Louis, MO 63110, USA; 3 Department of Pathology, St. Louis University Hospital, St. Louis, MO 63110, USA; 4 Department of Anesthesia and Critical Care, St. Louis University Hospital, St. Louis, MO 63110, USA

## Abstract

**Background:**

Extrapulmonary small cell carcinoma (EPSCC) involving the brain is a rare manifestation of an uncommon tumor type.

**Case presentation:**

We report a 59 year-old Caucasian female diagnosed with an EPSCC involving the left parietal lobe without detectable extracranial primary tumor followed by serial positron emission tomography/computed tomography (PET/CT) imaging. Histopathological examination at both initial presentation and recurrence revealed small cell carcinoma. Serial PET/CT scans of the entire body failed to reveal any extracranial [^18^F]2-fluoro-2-deoxy-D-glucose (FDG) avid lesions at either diagnosis or follow-up.

**Conclusion:**

Chemotherapy may show a transient response in the treatment of EPSCC. Further studies are needed to help identify optimal treatment strategies. Combination PET/CT technology may be a useful tool to monitor EPSCC and assess for an occult primary malignancy.

## Background

First described by Duguid and Kennedy in 1930 [1,2] EPSCC is recognized as a clinicopathologic entity distinct from small cell lung carcinoma [3-5]. Small cell carcinomas arising outside the lung have been reported in almost every organ of the body [5-7]. Primary locations include the head and neck, salivary glands, thyroid, larynx, trachea, thymus, pleura, esophagus, stomach, intestines, rectum, pancreas, gallbladder, cervix, uterus, breast, prostate, urinary bladder, and skin [8]. The most common site of presentation differs according to case series [1,6,7]. Only one case of an EPSCC involving the brain is documented in the literature [1].

An estimated one thousand new cases of EPSCC occur yearly, with an overall incidence between 0.1% and 0.4% of all cancers [4,9]. Approximately 2.5% of small cell carcinomas present at extraplumonary sites [3,4,8]. Since there is no national or international tumor registry, many cases are not reported, and the true incidence may be underecognized [9,10]. It is postulated that EPSCC originate from totipotent stem cells that can differentiate into various cell types [9].

The histologic criteria for EPSCC and small cell lung cancer (SCLC) are the same, namely uniform small cells with dense nuclei, inconspicuous nucleoli and sparse cytoplasm [10]. The presence of cytoplasmic argyrophilia or neurosecretory granules further substantiates the diagnosis [10,11].

Staging criteria for EPSCC is the same as that for SCLC. Limited disease (LD) is defined as a localized tumor with or without regional lymph node involvement; any extension beyond the loco-regional boundaries is defined as extensive disease (ED) [2,10].

Clinically these tumors represent a rare, heterogeneous group of neoplasms [12] characterized by their aggressive nature, early dissemination and propensity to recur [3,6,9].

Recent studies have demonstrated that extensive disease, poor performance status and an increased white blood cell count are the major prognostic factors that correlate with mortality [4,10].

Optimal management is not well characterized because of the rarity of these tumors, and the lack of randomized clinical trials to guide treatment; hence, there are no standard treatment regimens. Most of the data are extrapolated from treatment of small cell carcinoma of the lung. In this report, we present the clinicopathologic features and serial PET/CT evaluation of an EPSCC of the brain.

## Case presentation

We report a 59 year-old Caucasian female diagnosed with EPSCC of the left parietal lobe without evidence of an extracranial primary tumor. The patient presented with a three-week history of progressive deterioration of right upper extremity coordination and motor strength. A staging PET/CT scan and CT scans of the chest, abdomen, and pelvis, prior to surgery, were performed that revealed a left parietal lobe mass with an intense, FDG-avid rim anteriorly. Neither study showed evidence of metastasis in the lungs or elsewhere in the body. A magnetic resonance image (MRI) of the brain revealed a 4.4 × 4.2 × 4.5 cm left parietal mass.

The patient was treated with intravenous steroids and subsequently underwent an MRI-guided sterotatic left parietal craniotomy with complete resection of the tumor one day after admission. Samples of the resection were sent for pathological review. At diagnosis the complete blood count and complete metabolic profile were within normal limits. The patient did not experience either immediate or late post-surgical complications and was discharged to a rehabilitation facility for post-operative recovery and improvement of her performance status for future chemotherapy.

**Figure 1:**
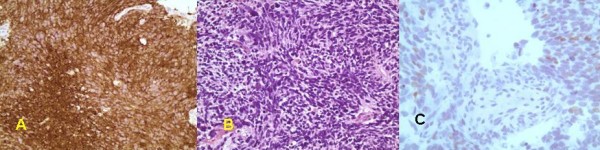
**Light microscopy and immuostaining of patient's tumor.** A) Syaptophysin immunostaining, 150×. B) Hematoxylin and eosin staining, 150×. C) Thyroid Transcription Factor-1 (TTF-1) immunostaining, 300×.

Pathologic examination of the parietal lobe resection was consistent with small cell carcinoma (figure 1). The tumor was reported as high-grade with nuclear pleomorphism, sparse cytoplasm and large areas of necrosis. The cells showed strong reactivity for synaptophysin and focally for thyroid transcription factor-1 (TTF-1). The tumor cells were negative for S-100, glial fibrillary acidic protein (GFAP), cytokeratin AE1/AE3 keratin, anti-cytokeratin (CAM 5.2) and chromogranin (figure 1).

Two months after initial resection surveillance MRI of the brain revealed recurrence of a left parietal tumor. The patient developed profound, progressive neurological deterioration consisting of hemiparesis and expressive aphasia. At this point she underwent a second complete resection of the tumor. Pathology again revealed a small cell carcinoma with an immunoprofile identical to that of the original specimen.

The patient experienced profound improvement in her neurological status after the second resection although her ECOG status was 2. She was discharged to a neuro-rehabilitation facility for recovery and optimization of performance status.

Four months following the second resection a 1.7 cm left parietal lesion was identified on MRI and chemotherapy with topotecan was initiated. This patient received six cycles of topotecan at the standard dose of 1.5 mg/m^2 ^on days 1 through 5 of a 21-day cycle. Overall, the patient tolerated therapy well with only grade 2 nausea and grade 2 anemia. Response to therapy was assessed by monthly MRI scans of the brain. After two months of therapy a reduction in tumor size greater than 50% was observed. The disease eventually progressed and was referred to hospice care 2 months following progression.

**Figure 2:**
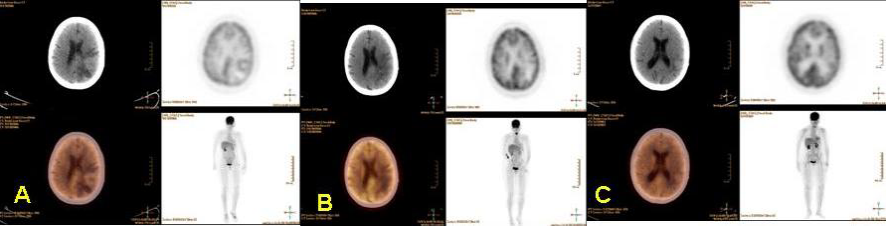
**No extracranial FDG-avid lesions to suggest malignancy are seen by PET/CT.** A) PET/CT, prior to craniotomy, showing anterior rim enhancement of the left parietal tumor. B) PET/CT revealing a photopenic area following second resection of the left parietal tumor. C) Follow-up PET/CT at 9 months.

On repeat PET/CT scans, following the second resection and 9 months after diagnosis (figure 2), there was absence of FDG-avidity in the left parietal lobe. Approximately one year after diagnosis of EPSCC, her disease progressed and the patient chose to enter hospice care.

## Discussion

The prognosis of EPSCC is similar to that of SCLC with fewer than 13% of patients surviving 5 years [10] although some patients have enjoyed prolonged survival and even cure [9]. Median survival for all patients has been reported to be 9.2 to 14 months [3,6]. The median survival for patients with LD is 19.8 months, while for patients with ED the median survival is 7 months [3]. The clinical course of this tumor is very aggressive, with a tendency for early systemic spread and recurrence after treatment.

Extrapulmonary small cell carcinoma is a rare, aggressive tumor for which there is no standard treatment guidelines [13]. Some authors suggest that optimal management of patients with EPSCC-limited disease consists of both local modalities (surgery or radiotherapy) and systemic therapy [10,14]. The chemotherapeutic regimens used for EPSCC are similar to those utilized for SCLC. Combination cisplatin and etoposide (EP) is a commonly used regimen for EPSCC with a response rates reported, in extensive disease, as 50% to 70%. It remains the cornerstone of therapy in SCLC [4,5,15]. In the first line setting single agent topotecan and paclitaxel have shown to be possible therapeutic options. Neither of these agents has been compared in a randomized phase III trial to EP [15]. Both surgery and radiotherapy have been employed for local control with varying degrees of success [3,10].

Since EPSCCs have responded well to agents active against SCLC, it was decided to initiate therapy in this patient. Topotecan was chosen because it was felt that the patient would not tolerate a platinum based regimen due to her poor performance status. Topotecan is a semi-synthetic derivative of camptothecin that specifically targets topoisomerase-I. It has shown clinical activity against SCLC [16]. The use of topotecan may be particularly appropriate for patients in which palliation of symptoms is the primary goal of therapy.

Immunohistochemistry can help diagnose EPSCC as these tumors stain positive for chromogranin A and TTF-1. Ordonez reported that TTF-1 lacks specificity to distinguish primary versus metstatic lesions [17]. Other studies have reported that TTF-1 may be useful in differentiating small cell from other extrapulmonary neuorendocrine tumors [18,19]. Extrapulmonary small cell carcinoma stains positive for TTF-1 while other extrapulmonary neuorendocrine tumors do not [18]. In contrast, a study by Prok demonstrated that in 16 of 43 patients with metastatic carcinoma of unknown primary to the brain, TTF-1 stained positive [20]. Positive staining for TTF-1 should be factored into each clinical setting when determining whether the tumor is an EPSCC or a metastatic lesion.

Whole-body PET imaging with FDG is used in the diagnosis, staging, and follow-up of many cancers with accuracies ranging from 80% to 90% [21]. PET/CT is still in its infancy; however, several studies published over the last few years demonstrate that PET/CT transforms image fusion from primarily a research tool to everyday clinical practice. In addition, these studies prove that PET/CT has a higher diagnostic accuracy than PET alone, or CT alone, or visually correlated PET and CT. Furthermore, PET/CT frequently provides statistically significant improvements over PET or CT alone in staging and restaging of different cancers [22]. Clearly, there are more data on the use of PET/CT in lung cancer than any other type of malignancy.

As the staging procedures for SCLC do not differ from those for non-small cell lung cancer (NSCLC) the primary role of PET/CT imaging is to delineate limited disease from extensive disease. There are relatively few indications for PET/CT scanning in SCLC as there is usually extensive disease at presentation. Nonetheless, it has been shown that whole-body PET is superior to conventional staging in the detection of all involved sites, thus it is a highly valuable tool for staging SCLC [23]. Further, dual-modality PET/CT is able to detect more primary tumors than PET, CT and PET and CT side-by-side in the diagnosis of carcinoma of unknown primary with less patient radiation exposure [24]. PET/CT can help localize the primary in CUP in approximately 40% of all cases, even after a thorough work-up with a variety of other investigations [25]. To the best of our knowledge, FDG PET/CT imaging of EPSCC involving the brain has never been reported before.

It is possible that the patient had an occult bronchial primary tumor that was beyond the limits of detection by PET/CT. There was an interval of nine months between the first and final PET/CT scans; this is a considerable amount of time for a bronchial primary to manifest. During this period the tumor recurred twice in the brain and serial PET/CT scans did not reveal extracranial malignancy at diagnosis or at later dates. As such we are of the strong opinion that the tumor did arise outside the lung.

## Conclusion

Our patient's response to therapy yielded a transient clinical response without profound toxicity. Further studies are needed to help identify optimal treatment strategies in this rare tumor type. Dual-modality PET/CT technology may be a useful tool to monitor EPSCC and may help in our understanding of this rare entity.

## Abbreviations

CAM 5.2: anti-cytokeratin; ED: extensive disease; EPSCC: extrapulmonary small cell carcinoma; FDG: [^18^F]2-fluoro-2-deoxy-D-glucose; GFAP: glial fibrillary acidic protein; LD: limited disease; MRI: magnetic resonance imaging; NSCLC: non-small cell lung cancer; PET/CT: positron emission tomography/computed tomography; SCLC: small cell lung carcinoma; TTF-1: thyroid transcription factor-1

## Competing interests

The authors declare that they have no competing interests.

## Authors' contributions

CH, NN, MO, NH, AP conception and design, acquisition of data, analysis and interpretation of data, have been involved in drafting the manuscript, revising it critically for important intellectual content and have given final approval of the version to be published.
